# Increasing community vulnerability to gastrointestinal infections in austerity’s shadow: a comparative study of two English local authorities

**DOI:** 10.1186/s12889-026-26657-1

**Published:** 2026-03-05

**Authors:** Suzanne Rotheram, Stephen Clayton, Dan Hungerford, Ben Barr

**Affiliations:** 1 National Institute of Health and Care Research, Health Protection Research Unit in Gastrointestinal Infections, The University of Liverpool, Liverpool, UK; 2 Department of Public Health, Policy and Systems, The University of Liverpool, Whelan Building, Liverpool, L68 3GB UK; 3 Department of Clinical Infection, Microbiology and Immunology, The University of Liverpool, Liverpool, UK

**Keywords:** Austerity, Gastrointestinal infections, Inequality, Environmental health, Infectious diseases, Vulnerability, Qualitative research

## Abstract

**Background:**

Gastrointestinal (GI) infections are spread through food, water and the environment. They cause significant morbidity and mortality worldwide and are a UK public health priority. In England, local authority (local government) Environmental and Regulatory Services (ERS) play a crucial role in GI infection services. During the period of UK government ‘austerity’ policies in the 2010s there were significant cuts to national spending, including reductions to ERS budgets which may have compromised GI infection prevention and control. In this paper, we examine how reductions in ERS spending across contrasting local authorities in England have shaped the lived experience of staff providing GI infection work, within the context of the COVID-19 pandemic.

**Methods:**

Data were collected between June 2021—March 2022 from staff linked to ERS across two urban English local authorities with contrasting ERS budget cuts, contrasting socio-economic deprivation and different local authority structures. Data consisted of observations (14 h) and semi-structured interviews (*n* = 17) collected from staff from the following teams: regional UK Health Security Agency teams (*n* = 6); local authority public health teams (*n* = 2); public protection teams (including Environmental Health Officers (EHOs)) (*n* = 6); and local authority-commissioned infection prevention and control teams (*n* = 2). Data were analysed using a reflexive thematic analysis.

**Results:**

Our analysis showed more significant impacts on GI infection prevention and control in our socio-economically ‘disadvantaged’ local authority, underpinned by a greater loss of EHO staff. Budget cuts in our ‘disadvantaged’ area have reduced the capacity for on-the-ground GI infection work in communities, hampered collaborative working across local health protection systems, and forced staff to ration and prioritise their work according to risk, reducing or discontinuing crucial preventative functions. Our ‘advantaged’ area was less affected.

**Conclusion:**

Contrasting levels of cuts have created socio-spatial inequalities in the experience of delivering GI infection prevention and control. Austerity may therefore be contributing to geographical inequalities in community vulnerability to GI infections. Staff should be supported in using risk assessment tools as they adapt their work practices to accommodate the impact of cuts. Illuminating these inequalities allows community vulnerability to GI infections to be more effectively targeted through policy measures.

**Supplementary Information:**

The online version contains supplementary material available at 10.1186/s12889-026-26657-1.

## Introduction

### Gastrointestinal infections: a public health priority

Gastrointestinal (GI) infections cause significant morbidity and mortality worldwide—spreading person-to-person, through the environment, through water, or via food [22]. In the United Kingdom (UK) one in four people experiences a GI infection each year, often accompanied by symptoms of vomiting and/or diarrhoea [22]. The two most common bacterial and viral GI infections, Norovirus and Campylobacter, are estimated to cost the National Health Service (NHS), and therefore the community, £16—£22 million each year and cost UK patients £114.6 million, through lost income, medication and childcare [58]. There is also increasing evidence of socio-economic and spatial inequalities in UK GI infections, particularly in the consequences of illness [47, 49, 51, 52]. Preventing and controlling GI infections is therefore a UK public health priority.

**Fig. 1:**
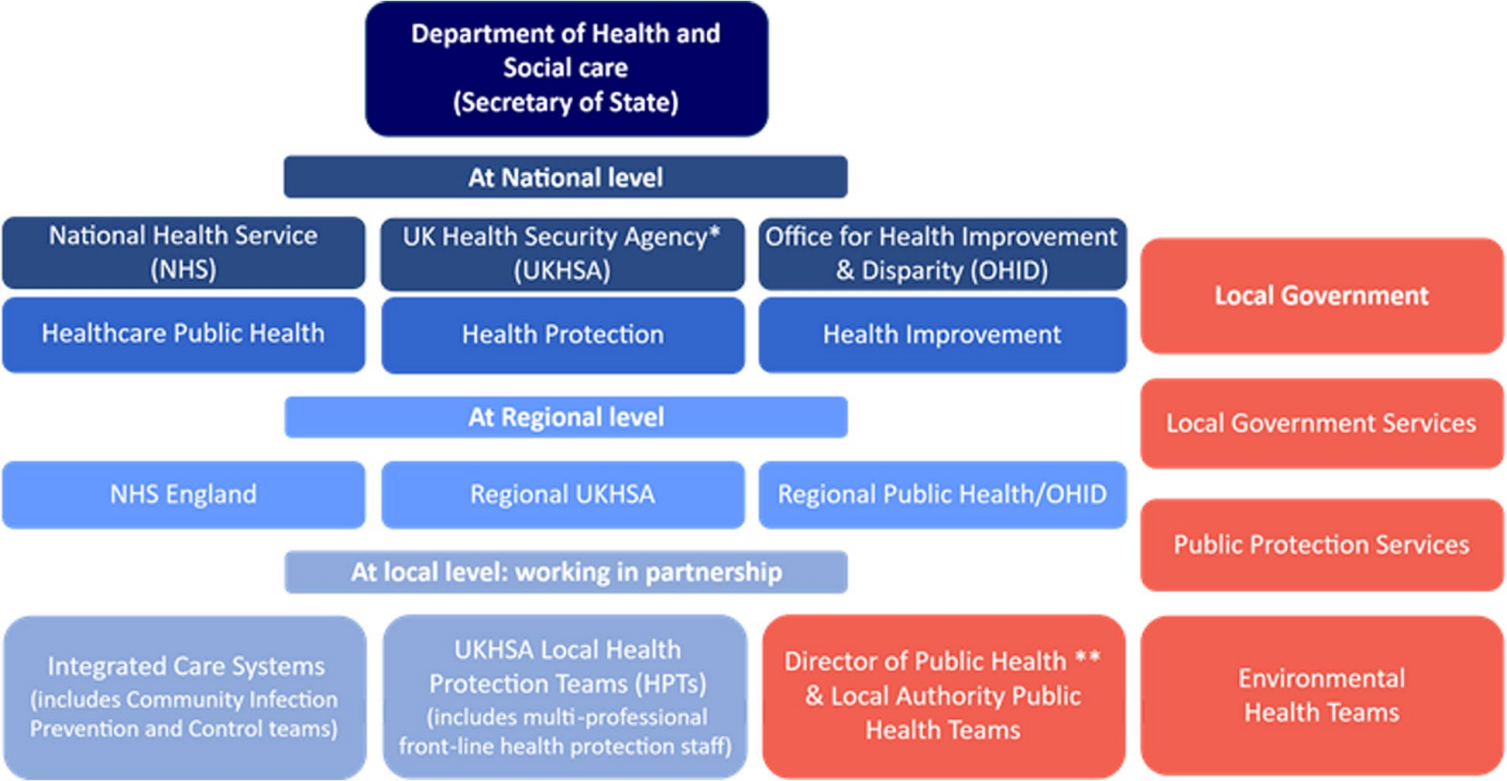
Public Health System structure (adapted from Fig. 1 [50]) * UK Health Security Agency was formally Public Health England (PHE). ** Accountable to the Secretary of State on public health protection and health improvement functions delegated to Local Authorities

Multiple organisations are instrumental in mitigating the social and economic impacts of GI infections within local communities in the UK. In England, these organisations sit within a larger Public Health System but work in partnership at a local level in ‘local health protection systems’ (see Fig. 1). Within these local health protection systems, professionals with responsibility for local GI infection prevention sit across four main organisations: the UK Health Security Agency (UKHSA), Public Protection Teams, Local Authority Public Health Teams and Community Infection, Prevention and Control Teams (Fig. 1). The UK Health Security Agency (UKHSA) is the national non-governmental body with oversight for public health in England and operates at a national, regional and local level. Public Protection Teams sit within local government Public Protection Services and deliver Environmental and Regulatory Services through Environmental Health Teams. Local authority public health teams also sit within local government and are responsible for local authority public health services. Lastly, Infection, Prevention and Control Teams are responsible for managing outbreaks of infectious diseases in community settings. These teams sit within the National Health Service (NHS) Integrated Care System and in some areas are commissioned or delivered by local authorities [23].

Since the introduction of UK government austerity measures in 2010, there are concerns that these local health protection systems, and their effectiveness, are being compromised [6, 60, 61]. Moreover, research increasingly points to the unequal geography of austerity’s impacts [2]. Yet to date, there has been little research examining the impact of austerity on GI infection prevention and control in different places. This paper aims to fill this gap by exploring the connection between austerity, the provision of GI infection prevention and control and place.

### Linking GI infections, austerity and place

‘Austerity’, meaning ‘severe restraint’, refers to national policies to cut spending or raise taxes, usually to reduce government debt, especially in response to the effect of recession on government finances [55, 62]. In the UK, the Conservative Party and the Liberal Democrats' coalition introduced austerity policies in 2010 to reduce government debts that had resulted from the 2008 financial crisis [18, 46]. UK austerity policies have focused on cutting spending through substantial cuts in welfare protection and local government (termed local authorities in England) budgets [54]. As a large proportion of local authority income comes from central government grants rather than local taxation [31], English local authorities were particularly exposed to national austerity measures; consequently, between 2010–2017, local authority budgets in England were cut by 50% [43]. Austerity is therefore highly pertinent to our study given that local authority Environmental and Regulatory Services (ERS), delivered by local authority public protection teams, are central to GI infection prevention and control and have been substantially impacted by these budget reductions.

**Fig. 2:**
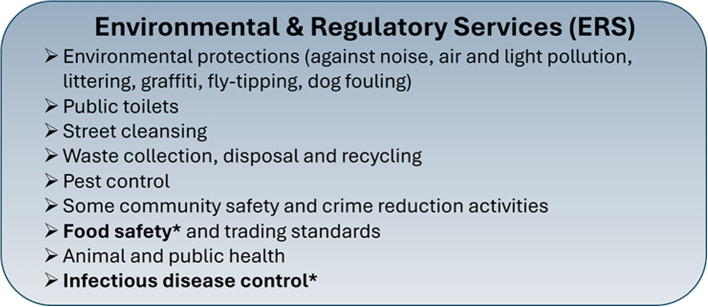
Overview of environmental and regulatory services. Adapted from Fig. 1. [40] *Highlighted activities related to GI infection prevention and control

Environmental and Regulatory Services are delivered by public protection teams who employ Environmental Health Officers (EHOs) to provide many of their services. EHOs statutory duties span food hygiene and safety, private sector housing, environmental protection, health and safety at work, public health, food standards and licensing [15]. In this paper we focus solely on EHO work directly related to GI infection prevention and control, specifically food safety and infectious disease control which fall within ERS (Fig. 2, highlighted activities). Key EHO activities in this area include inspecting food premises, taking food samples, investigating food-borne GI infections (including outbreaks) and enforcing food safety standards [9]. The potential impact of EHO work on GI infections is extensive. In 2020, EHOs in England and Wales inspected over 562,000 food premises, took 156,000 formal actions to improve food hygiene and dealt with 58,434 incidents of notifiable infectious diseases [36].

Between 2009–2018 austerity policies reduced ERS funding, per head of population, by 53% [6, 60]. This led to a 56% decline in professionally qualified food standards staff numbers and a 16% reduction in food hygiene staff [6]. However, the scale of these cuts have not been equitably distributed across local authorities. Spending per capita on ERS between 2009/10 and 2020/21 declined by 22.8% in the most ‘disadvantaged^1^’ areas compared to 6.3% in more ‘advantaged’ areas [42]. These unequal impacts also appear to be shaped by local government structures. In England some areas have 2 tiers of local government, with responsibilities split between upper and lower tiers (known as county councils and district councils). In two tier areas ERS are the responsibility of the lower tier. Other areas only have a single tier of local government which is responsible for all local government services including ERS. ERS services have tended to be cut more in single tier areas, where funding has been redirected away from ERS to protect statutory social care services—which account for the majority of expenditure for single tier authorities. [31, 42]. As a result, ‘disadvantaged’ and single tier local authorities have seen greater cuts. Austerity therefore has the potential to incur unequal, unjust impacts on GI infection work, and health, across different areas of England, something already recognised in other place-based literature outlined below.

### Connecting health, austerity and place

A growing body of evidence indicates that austerity measures worsen health outcomes in the countries that implement them. For example, the UK and Greece, which have imposed severe austerity-driven budget constraints, have experienced a recent deterioration in mental health, suicide rates, life expectancy, alcohol and drug misuse, infant mortality, HIV and tuberculosis [5, 8, 35, 62]. In contrast, countries which did not introduce austerity policies over the same period, have seen fewer deleterious changes to population health [5, 62].

As well as disparities *between* countries, there is evidence of geographical differences in the experience of austerity *within* countries [7, 54]. In the UK, for example, as central government grants make up a larger share of income for councils in areas defined as ‘disadvantaged’, these councils have been disproportionately affected by cuts [29, 31]. In the UK, ‘disadvantaged’ local authorities have experienced cuts nearly six times greater than ‘advantaged’ areas in funding allocation for all services (excluding education and public health) [29]. These differential cuts, have, in turn, been linked to higher rates of mortality and poorer physical and mental health in areas of higher socio-economic disadvantage [2, 3, 8]. These clear inequalities in health between different places and socio-economic groups have been described as *‘systematic differences in health status between different socioeconomic groups [that are] socially produced (and therefore modifiable) and unfair’* [65].

Although the literature exploring the impact of austerity between places is expanding, to our knowledge, no research has examined the impact of contrasting ERS cuts on the lived experience of providing GI infection work. This is particularly concerning considering increasing evidence for inequalities in ERS budget cuts according to both levels of disadvantage and local authority structure [42]. We set out to fill that gap by examining if contrasting ERS cuts are shaping the lived experience of local health protection staff linked to ERS services in England. It is important to note that interviews were carried out during the COVID-19 pandemic, and we have explored the effect of austerity on the management of COVID-19 by local health protection systems elsewhere [50].

We frame our paper by drawing on the concept of ‘vulnerability’. Within GI infection research, vulnerability is used almost exclusively to refer to individual biological factors (e.g. age) or individual behaviours (e.g. hygiene practices) which interact with a person’s exposure to a pathogen and increase their susceptibility to illness [32, 38]. In this paper, we draw on a wider sociological conceptualisation of vulnerability which, ‘can contain a multitude of factors (biological, social, or otherwise) influencing ill health’ [45]. This broader conceptualisation allowed us to recognise that, consistent with the social determinants of health model [17], it is not only biology or individual behaviours that determine vulnerability to infectious diseases but also the interconnectivity between infectious diseases and wider social and structural factors found within places. In so doing our study aimed to: *examine the locally situated prevention and management of gastrointestinal infections by local health protection systems in contrasting local authorities, linking it to their social, environmental, economic and policy contexts.* We do this by comparing data collected from two contrasting areas, described in more detail in the methodology section next.

## Methodology and methods

### Choosing contrasting study areas

To compare GI infection work across two contrasting local authorities we chose study areas with contrasting levels of budgets cuts, deprivation and local authority structures, because existing literature had already demonstrated that these parameters were linked in the patterning of austerity’s impact on other local authority services [29]. In so doing we hoped to draw out place based, socio-economic inequalities in the impact of austerity on ERS. Additionally, consultation with a group of EHOs revealed that the structure of local authorities was a key area of professional interest, particularly as an increasing number of local authorities moved from two-tier to single tier structure [53]. Our two study areas therefore, had different local authority structures (1 tier vs 2 tier), contrasting deprivation (20–25% of population income deprived vs 5–10% of population income deprived), and contrasting reductions in ERS spending since UK austerity measures were introduced in 2009 (−39% vs −25%) (Table 1). The local authority with a 39% reduction was in the top 20% of local authorities with respect to cuts and the local authority with a 25% reduction was in the bottom 60% of local authorities with respect to cuts.

**Table 1:** Overview of the two local authorities included in the study

	Area (pseudonym)	
	Stoneville	Meadowshire
Type of Local authority^a^	One-tier	Two-tier
Urbanicity	Urban	Urban
Population^b^ (rounded to the nearest 50 000)	500 000	150 000
Population density^b ^ (usual residents/km^2^) (rounded to the nearest 500)	4 500	500
% of population income deprived^b^	20—25	5—10
Population Ethnicity (%) White British^b^	75—80	75—80
% change Environment & Regulatory Services expenditure^c^ (per capita 2009—2017)	−39	−25
Number of Environmental Health Officers (Full-time equivalent)^d^	9	6.5
Number of food businesses^d^	4000	1500
No. of professionally qualified staff per 1000 food businesses	2.25	4.3

^a^Some parts of England have 1 tier of local government some have 2. In one-tier areas, all local services are funded from the same budget. In two-tier areas, ERS is the responsibility of the lower tier [31]

^b^Figures from UK Census ONS data 2021 [44]

^**c**^ERS data taken from [1]

^**d**^Numbers as reported by participants at the time of data collection (see results)

We call the ‘disadvantaged’ local authority with higher cuts to ERS expenditure *Stoneville*, and the ‘advantaged’ area with lower ERS cuts *Meadowshire* (Table 1). We use pseudonyms to protect the anonymity of our participants working in small teams and so that participants felt able to talk about the impact of government policies without fear of repercussions.

### Methodological approach

Ethical approvals for the study were received from the University of Liverpool and public involvement was integrated throughout [57] (Appendix A). The study drew on the tenets of an ethnographic approach and so used observations and interviews to gain insights into what local health protection teams do, how they understand these actions, and how they fit into the social, economic, and policy context in which they work [28]. In practice, non-participant observations (i.e. the researcher observed but did not take part in the activity) were conducted by SR with EHOs and focused on observing their normal working activities [64]. Semi structured interviews were utilised so we could examine in-depth experiences and practices related to GI infection work with respect to austerity with participants [27]. Interviews were conducted by SR with staff working in different parts of the local health protection system and focused on understanding the role of participants managing GI infections and if or how their roles had been affected by budget cuts.

Before starting recruitment, the study team contacted the Directors of Public Health in both study areas to gain approvals and assist with introductions to managers of public protection teams. To mitigate researcher selection bias, managers of the public protection teams informed their team about the study so that all EHOs with roles related to GI infections understood why the research was being conducted and had the opportunity to contact the research team and take part. Once EHOs had been recruited, snowball sampling was then used to recruit other professionals with links to EHOs ERS work. Participants were therefore UK Health Security Agency (UKHSA) staff (*n* = 6), public protection team (PPT) staff (*n* = 6), local authority public health team (LAPHT) staff (*n* = 3), and Infection, Prevention and Control Team (IPCT) staff commissioned by local authorities (*n* = 2) (Table 2). Participants gave written, informed consent to take part.

**Table 2:** Pseudonymised participant information table

Pseudonym	Area	Team
Sarah	Stoneville	Public Protection
Michelle	Stoneville	Public Protection
Gary	Stoneville	Public Protection
Rachel	Stoneville	Local authority public health
Amy	Stoneville	Regional UKHSA*
Elizabeth	Stoneville	Regional UKHSA
Ruth	Stoneville	Regional UKHSA
Thomas	Stoneville	Infection Prevention and Control
Louise	Meadowshire	Public Protection
Andrew	Meadowshire	Public Protection
Miranda	Meadowshire	Public Protection
Alistair	Meadowshire	Local authority public health
Tracy	Meadowshire	Local authority public health
James	Meadowshire	Regional UKHSA
Sally	Meadowshire	Regional UKHSA
Hannah	Meadowshire	Regional UKHSA
Philippa	Meadowshire	Infection Prevention and Control

PHE ceased to exist in 2021. Participants therefore identified either as being part of PHE or the UKHSA, depending on when they were interviewed. For clarity in this paper (except when it is being used in a direct quote by participants) we use the more recent term – the UKHSA

^*^UKHSA = UK Health Security Agency (formally Public Health England (PHE))

Data collection took place between June 2021 and March 2022. This coincided with the end of the COVID-19 pandemic response which had seen a marked decrease in GI disease reported to health services in England [37, 59]. During data collection for this study, gastroenteritis outbreaks and laboratory reports of GI pathogens had increased but to levels lower than pre-COVID-19 pandemic averages [59]. Data collection included: 14 h of fieldwork observations and informal conversations collected while observing EHOs recorded using fieldwork notes, and 17 semi-structured interviews (Meadowshire (*n* = 9), Stoneville (*n* = 8)) lasting around 1 h, conducted online with the PI and participant present, using Microsoft Teams. The semi-structured interview schedule was developed for this study and is published in our paper exploring the effect of austerity on the management of COVID-19 by local health protection systems [50]. Interviews were recorded and fieldnotes and interviews were transcribed.

Semi-structured interview data and fieldnotes analysed for this paper focused on participants’ GI infection role and if or how budget cuts had shaped their work. Data analysis was informed by an inductive, reflexive thematic analysis across the whole dataset carried out in NVivo 12 [14]. The analysis involved an iterative process between data familiarisation, assigning ‘codes’ (units of meaning) to the data, grouping codes into themes with shared patterns and meaning, and refining coding so that it was internally coherent [13]. In practice, SR and SC led the data analysis with SR coding all the transcripts and discussing the coding with SC. SR and SC then refined the codes and developed themes. These themes were then discussed among the whole research team and further refined. Any disagreements were resolved by discussion (Appendix B). Analysis paid particular attention to contrasts across Meadowshire and Stoneville so that differences in the impacts of budget cuts could be drawn out.

In our following results we first present data that contextualise the impact of contrasting ERS budget cuts for public protection teams in Meadowshire and Stoneville. We then present three themes which we identified across our dataset as being crucial facets of local health protection systems and effective GI infection work. Within each theme we compare the impact of cuts on this aspect of GI infection work in Meadowshire and Stoneville. Our first theme describes the impact of austerity on the physical presence of public protection teams within communities. Our second theme shows how budget cuts have shaped aspects of collaborative working across local health protection systems. Our third and final theme illustrates how austerity has changes different aspects of risk management in the prevention and management of GI infections within our study areas.

## Results

### The context of the cuts in Meadowshire and Stoneville

Meadowshire and Stoneville, our study sites, are urban areas with contrasting local authority structures, levels of ‘deprivation’ and levels of ERS cuts (Table 1). Participants recognised that their experiences of budget cuts were shaped by the relative ‘deprivation’ and structure of the local authorities in which they worked.

Michelle explained that being a ‘disadvantaged’ local authority had led to Stoneville losing proportionally more income from central government grants than more ‘affluent’ local authorities who received higher income from residential and business rates. In addition, as a single-tier local authority, Stoneville had to use this reduced income to provide education and social services as well as ERS. Within this context, her public protection team had lost out:

*… because it is quite an impoverished area, we [Stoneville] don’t get the income from the [residential and business] rates (…) And also there is, a huge want for social services, we have got a lot of adult services that take a great deal of funding… and education… (…) so ourselves in public protection we are probably not seen as that significant compared with a child being abused and having to deal with that. So a lot of that has taken priority (…) I think that is why we have lost out in the resource…* (Michelle, PPT, Stoneville).

In contrast, Meadowshire was classified as being in the least ‘deprived’ fifth of local authorities in England and had therefore lost proportionately less income from central government residential and business rates (taxes). As central government grants make up a larger share of income for councils in areas defined as ‘disadvantaged’, these councils have been disproportionately affected by cuts [29, 31]. Louise recognised that her ERS budget had been further protected because Meadowshire’s two-tier (district) local authority did not have to fund social services or education from the budget that also funded ERS:

*…. that is a big difference between a district council [two-tier] and a metropolitan council [single-tier], in as much as social care comes within their budget, whereas we don’t have to do education and we don’t have to do social care from the district council budget (…). So from that respect I know [metropolitan council] colleagues—their funds have been cut more significantly than the district council funds….* (Louise, PPT, Meadowshire).

These contrasting contexts led to stark differences in budget cuts. Between 2009–2017 Stoneville’s ERS expenditure per capita reduced by 39% (a £93 reduction per capita) compared to a 25% reduction in Meadowshire (£34 per capita) [1].

In turn, cuts were directly linked to reductions in EHO staff. Michelle explained that after austerity measures were introduced in 2010 her team had, at one point, been reduced from 26 to 6 EHOs:

Michelle: *Since 2010 there has just been dramatic cuts. I was actually on [long-term] leave in 2010 and when I came back I think half the staff had gone, and really quickly, like virtually overnight (…) And it wasn’t just in 2010 it continued and has continued (…) I think it went down to 6 EHOs at one point*.

Interviewer*: … what kind of numbers were you looking at before these cuts happened?*

Michelle*: We were about 26 officers.* (Michelle, PPT, Stoneville).

At the time of data collection in 2021/2022, Stoneville had 9 EHOs (including one trainee) responsible for 4000 registered food businesses, making 2.25 EHOs per 1000 businesses.

In contrast, Louise described Meadowshire’s loss of staff as, *‘3 or 4 people in the last 10–15 years’*. Meadowshire had therefore seen a less dramatic drop from 10, to 6 EHOs working full-time and one working part-time, covering 1500 food businesses. Meadowshire therefore, had 4.3 staff per 1000 businesses, nearly twice as many as Stoneville’s 2.5 staff per 1000 businesses.

These contrasting staff losses provide an important context for understanding our three themes below. Within each theme, which depict different aspects of local health protection system GI infection prevention and control, we first demonstrate why they are a necessary part of GI infection work and then show how ERS cuts have shaped the lived experience of delivering this work, comparing experiences across our two study areas.

### Theme 1: boots on the ground

The first facet of health protection identified in our data as being a crucial component of effective GI infection work was the presence of public protection staff as ‘boots on the ground’, by which we mean being physically present in communities. In practice, in relation to GI infection work, this meant firstly, that EHOs were relied on for their onsite expertise – specifically to inspect food premises in-person to prevent foodborne illness, and secondly, that EHOs were accessible to the public and able to respond to public concerns regarding food safety.

#### Onsite expertise: physical inspection of food premises

The importance of EHOs physically inspecting food premises to prevent GI infections was illustrated in fieldnotes from Stoneville where Sarah, an EHO, was observed inspecting a café:

*Sarah picked up her red clipboard, attached the inspection documentation [where she recorded her observations] and walked to the back of the café. She started opening fridges, describing her findings to me as she went along. She found holes in walls which rats and mice might be able to get through, dirty floors, grease around the frying area, leaking sinks, uncovered and un-dated food in fridges and freezers, a toilet with no soap and food in the cold storage area that wasn’t cold because the unit wasn’t plugged in (…) She showed me that the electric fly trap in the front was full and that fly paper had been positioned over food. (…) she then asked the person who was in charge if she could see the paperwork [which recorded evidence of temperature checks and pest contractor visits] (…) the records only went back to 2020 and no records were found of temperature checks.* (Fieldnotes, Stoneville).

The physical inspection which Sarah conducted, described above, identified multiple ways in which this café’s storage and preparation of food had the potential to make people ill—uncovered food, no record of when food should be consumed, access points for rodents, food stored at the wrong temperature, fly traps positioned over food, and no recent record of temperatures or pest checks. By finding these risks, recording them, and then doing a follow-up visit to make sure they were addressed, Sarah was protecting staff and customers eating at that café from practices that might lead to foodborne illness.

A loss of staff due to budget cuts had reduced the capacity of public protection teams in both local authorities to carry out inspections of food premises. This had been compounded by the COVID-19 pandemic when in-person inspections were not allowed. The following extract shows how despite the COVID-19 backlog, EHOs in Meadowshire had been able to keep on top of their inspections, but weren’t sure if this would continue:

*Just before I left, they [Louise and colleagues] were talking about the backlog of inspections they had due to COVID-19. Louise printed out a 4-page list of businesses she needed to inspect. She said that the team had just managed to inspect all the ‘A’ businesses [the highest priority] and had to do the ‘B’ businesses by June, which she thought was just about doable. They then had to do the ‘C’ businesses by September—she wasn’t sure they would get done (Fieldnotes, Meadowshire).*

In contrast, the public protection team in Stoneville described having a backlog of inspections even before the COVID-19 pandemic. Gary explained that their current staffing levels did not allow them to keep on top of their statutory (legally required) inspections of food premises:

*… [the number of EHOs] is still nothing compared to how it used to be …. we are way below what we need to be to enable us to comfortably deal with our statutory requirements with the number of premises we have….* (Gary, PPT, Stoneville).

#### Accessibility to the public: responding to public concerns

The second way EHOs worked as ‘boots on the ground’ was by being accessible to the public and able to respond to complaints or requests for advice. Examples in fieldwork included EHOs receiving reports from the public of alleged food poisoning incidents, giving advice to members of the public during outbreaks of infectious diseases, and providing advice on safe food preparation for public events.

The accessibility of EHOs to the public was particularly important because while other parts of the local health protection system directed much of their advice to partners, EHO work was more public facing, as Louise described here:

*…. generally, we are the horse's mouth so to speak, so if people have food hygiene concerns or want advice about what they should be doing, they tend to come to us …* (Louise, PPT, Meadowshire).

Despite the clear importance of this aspect of GI infection work, participants in other parts of the local health protection system perceived that cuts had reduced the capacity of EHOs to respond to public concerns. Ruth, who worked for the UKHSA said that budget cuts and a loss of EHO staff meant that EHOs were:

*… not following up on cases as much, not following up on complaints as much. (…) So they have not got the staff. So there are lots and lots of things going under the radar for sure… (Ruth, UKHSA, Stoneville).*

This observation from Ruth was evident in Stoneville’s EHO team, who, as Sarah explained, did not respond to all complaints, but prioritised the ones which were ‘risky’ i.e. more likely to be significant to public health:

*An awful lot of complaints we don’t follow up, we only follow up the very risky ones (…) so things that would actually physically cause harm or would be significant [to] public health…* (Sarah, PPT, Stoneville).

In contrast, fieldnotes showed that the EHO team in Meadowshire were able to follow up on all their complaints:

*I asked [an EHO] if he followed up on all their public complaints. He explained that they do because they are employed by the local authority, and it reflects badly if they don't follow up on the complaints of people in their local area.* (Fieldnotes, Meadowshire).

Our data therefore shows that EHOs were integral to GI infection work by being ‘boots on the ground’ but our evidence indicates that budget cuts were compromising their capacity to inspect food premises and respond to public concerns, particularly in Stoneville.

Next, we look at how local health protection system integration was essential to effective GI infection control, and the impact of cuts.

### Theme 2: local health protection system integration

Participants recognised that to provide effective GI infection work, teams within local health protection systems (i.e. public protection teams, UKHSA teams, local authority public health teams and infection prevention and control teams) with a range of specialist skills and expertise needed to work together. Phillipa explained that without this collaboration, she couldn’t do her job:

*So I think in order to keep abreast of everything you have got to keep those relationships [with other teams] going. Because [otherwise] it just won’t work (….) we do all work collaboratively together no one really is on their own*. (Phillippa, IPCT, Meadowshire).

Reliance between different parts of the system was particularly evident in descriptions of the successful management of GI infection outbreaks. In these situations, as James explains here, outbreak control teams (a group of people with contrasting skills and expertise) were formed between the UKHSA, EHOs and other partners:

*We have always had very, very, very close links with the environmental health departments because for GI outbreaks they are a core member of the outbreak control team.* (James, UKHSA, Meadowshire).

Amy’s description of a GI outbreak investigation attributable to contaminated oysters illuminates how the specialist skills and knowledge of the UKHSA, who collated and analysed information on what people had eaten, were used alongside EHOs on-the-ground access to food premises and knowledge of how food should be safely stored:

*… between ourselves and the EHOs we managed to pin it down to… it was the oysters that had caused them to all be sick, but it is a very joint approach because the EHOs won’t have had that knowledge of what [people] have eaten as individuals, but likewise we haven’t got eyes on the ground to be able to say well there is a problem with the cold chain storage (…) So we are very dependent on each other…* (Amy, UKHSA, Stoneville).

Historically, EHOs working in local authorities developed specific areas of expertise required for investigating GI infections by being assigned to specialised teams. Sarah described how one consequence of budget cuts in Stoneville was that these teams had been merged:

*I started ID [Infectious Disease] work in [Stoneville] and that was my full-time role. And there would have been maybe 4/5 people on the team then, so our role then would be purely investigation of food poisoning cases (…) But over time, and then since budget cuts basically that has all fallen away. So we had an ID team, we had a food team, we had a health and safety team, but over the years they were just all merged together.* (Sarah, PPT, Stoneville).

Sally explained that this loss of EHOs with specialist skills meant that she, and her UKHSA colleagues, were being called on more often to help less experienced staff:

*… what we have seen is perhaps the need to support much more than in the past when you had very experienced knowledgeable staff, senior staff …* (Sally, UKHSA, Meadowshire).

In contrast, Meadowshire’s EHO team still had the capacity, skills and experience to support colleagues from other areas, as this quote from Michelle shows:

*They [Meadowshire’s EHOs] are a great team, they are very open and proud of what they do so they will happily share any of their knowledge and policies and procedures, with any other councils (…) And you don’t see that so much these days and it is because there is just not the boots on the ground with the other councils so we are in a fortunate position I think*. (Miranda, PPT, Meadowshire).

This theme demonstrates how a loss of EHO staff with specialist skills had reduced some aspects of collaborative GI infection work and, consequently, put pressure on other parts of the health protection system.

In our final theme, we explore the relationship between budget cuts and GI infection risk.

### Theme 3: managing ‘risk’

Within health protection, assessments of ‘risk’^2^ are routinely used to decide the potential for harm to the population and what kind of response is required [23, 30]. Our final theme shows how austerity has shaped two different aspects of risk. First, we show how assessments of ‘risk’ are increasingly being used to prioritise work within the context of limited resources and staffing. Second, we show how this prioritisation is leading to a loss of preventative work, particularly in Stoneville.

#### Using assessments of ‘risk’ to prioritise work

Participants gave multiple examples of how assessments of risk were routinely used to prioritise GI infection work. Sometimes formal protocols were used to make these decisions, as Michelle described here:

*… we do have set protocols. There is a large document about what diseases we all look at and response times, some have a 24-h response time, some are a 48 [hour] response time, some are only questionnaire, some are visits (…) it depends on how severe the illness is.* (Michelle, PPT, Stoneville).

At other times, Amy explained that experience and judgement were used to decide what to respond to, when, with more resources, health protection teams would ideally be responding to everything. As Amy noted:

*… you are almost constantly having to risk assess in your head—I have got three outbreaks going on here, there is only one of me to provide input or support, which one am I going to give the priority to? Which one as a system are we going to give the priority to? And of course in an ideal world you would give priority to absolutely everything but a viral norovirus in a nursery, would take much less precedence over, an STEC [Shiga toxin-producing E. coli] outbreak, it is going to have to ….* (Amy, UKHSA, Stoneville).

Participants in Meadowshire did not give any specific examples of budget cuts changing the way they prioritised work within Environmental Health Teams. In Stoneville, however, it was clear that a loss of staff had led to the Environmental Health Team being forced to give precedence to work considered to be ‘high-risk’, while other activities under their remit had to wait:

*… if you shrink down the core of your business all you tend to look at are things like, very severe accidents, you would look at fatalities, you would look at the high-risk GI infections such as STEC, you would look at places where you think they have got pests and where there might be an imminent risk to health and it needs closure (…) So it is all to do with severity of either illness or it depends on severity and that is what we look at. And everything else has to wait.* (Michelle, PPT, Stoneville).

This shows how, as budgets had shrunk, the importance of risk assessments had increased. This had repercussions on other aspects of GI infection work as focusing only on ‘high risk’ functions meant that other important areas of work were delayed or discontinued. We describe what this looked like in the context of preventative GI infection work below.

#### Preventative work

The second way that participants managed risk was through their preventative work. In an ideal world this would have been a priority for all participants, as indicated by Tracey in her quote below:

*I think with all of the workstreams that I am responsible for, we would like to think we are trying to get upstream*^3^* [by which she meant working towards prevention] as much as we can at the same time as dealing with the reactive stuff.* (Tracey, LAPHT, Meadowshire).

Participants gave multiple examples of their work which contributed to preventing GI infections, including: food hygiene inspections; food preparation advice; care home infection control inspections and food sampling surveys (described next).

One crucial aspect of preventative work carried out by EHOs was food sampling surveys and environmental swabbing. These activities involved selecting food premises at random and visiting them in-person to take samples of food and environmental swabs to check levels of bacteria. Louise demonstrated the importance of this work by describing a routine sampling programme where she found a contaminated radio with the potential to make people ill:

*So, I remember going into a hotel and we swabbed the radio that was shared between a pastry area and a raw meat area and the chefs would press the button on this horrible manky radio in the middle of the kitchen [which] was really grim and was crawling with bugs. I am surprised we didn’t see them (laughs). It came back really quite shocking as to oh right, you have got this time bomb in the middle of your kitchen.* (Louise, PPT, Meadowshire).

Despite the desire of participants working across the local health protection system to focus on preventative work, multiple participants gave examples of their teams being forced to prioritise statutory or reactive work over their preventative functions. Rachel gives one such example here, describing how Stoneville’s EHO team had been forced to stop much of its preventative work in the community:

*…we had a bit of a learning hub a few years ago looking at work with food-related work, and they [EHOs] had done lots of work in the past around prevention and education, and different schemes but [since cuts] they were very much having to just focus on their statutory role and not really able to deliver that either.* (Rachel, LAPHT, Stoneville).

One significant impact of budget cuts was a drop in participation in food sampling surveys. Hannah acknowledged that not all EHO teams had historically taken part in sampling programmes, but that EHO staff losses had reduced food sampling further in many of the health protection teams across her region, giving an example here of what this looked like in practice in one city council she worked with:

*… [city council] for example, they [used to have] three dedicated food sampling officers and they just don’t have any dedicated food sampling officers now, their team have shrunk hugely…* (Hannah, UKHSA, Meadowshire).

The repercussions of being able to take part in fewer food surveys were, in Ruth’s opinion, that local health protection systems were: *‘potentially missing outbreaks.’* (Ruth, UKHSA, Stoneville).

In our data we found clear contrasts in the participation in food sampling surveys between our two study areas. Louise described her team in Meadowshire being involved in food surveys for ‘*cooked meats, salads, ice from ice machines in pubs and ice creams*’. She acknowledged that with fewer staff her team had reduced their involvement in sampling projects, but said they still routinely participated in this work:

‘*….normally we would be out most months taking routine food samples.’* (Louise, PPT, Meadowshire).

In stark contrast, Stoneville’s EHOs could not take part in routine sampling programmes. This was because, as Gary explained, their sparse resources had to be used to prioritise their statutory functions – food premise inspections:

*So in effect, what has happened is, that we have been forced down a road where we are constantly chasing our tail just to meet our statutory requirements and we have had not the benefit of doing, or the luxury to be able to invest time to do, other preventative work.* (Gary, PPT, Stoneville).

Inequalities in the management of risk were therefore evident between Meadowshire and Stoneville. Meadowshire had been able to retain some preventative work, whereas Stoneville was increasingly being forced to use assessments of risk to prioritise their work and had ceased most non-statutory preventative functions. We now discuss the implications of these differences, alongside our other findings.

## Discussion

### Inequalities in the provision of GI infection services

This study has examined the lived experience of austerity for staff linked to GI infection work in two contrasting local authorities. It shows that while budget cuts have shaped GI infection work across both local authorities, their impact on the lived experience of participants working in our ‘disadvantaged’ local authority, Stoneville, was greater than in our ‘advantaged’ area, Meadowshire. Some of our findings, such as reductions in preventative work and food business advice, or struggling to keep on top of statutory inspections, are consistent with other reports exploring the impact of budget cuts on Environmental Health [15, 16, 29, 60, 61]. We add to this existing literature by showing how contrasting ERS cuts due to austerity can lead to distinct geographical differences in service provision, in terms of in-person EHO activities, collaborative working, prioritisation of services and preventative work. Recent quantitative research has demonstrated that lower ERS spending per capita leads to reduced EHO staffing and food inspections [41], our study expands on this work by showing how these staff losses are enacted in other everyday practices across local authorities with contrasting cuts.

The inequalities in ERS provision that we find in our study resonate with literature linking austerity to other aspects of health and societal conditions [4]. Austerity has been blamed for increasing infectious disease rates and mortality for marginalised populations during the COVID-19 pandemic [39]. In the longer-term, austerity measures have been linked to increased food bank usage and food insecurity, worsening mental health, declining mortality rates, a lowering of life expectancy and increasing numbers of children being taken into care [2, 4, 8, 10, 11, 33, 62]. Our study extends this already broad evidence base by showing how austerity might impact GI infections and could be considered a social determinant of inequitable health outcomes in GI infections – by creating unjust and unfair differences in services across socio-economically contrasting people and places [4, 17].

Importantly, our findings show that these unequal impacts go beyond inequalities in losses in staff and their activities to include a loss of ‘soft’ intelligence from interactions with communities and reductions in prevention activities which potentially further increase workloads and exacerbate resource constraints. Evidence suggests that the costs of austerity (in terms of excess deaths, years of life lost, etc.) significantly outweigh any fiscal savings achieved through reduced public expenditure [11] More research is needed to determine whether reductions to ERS undertaken in pursuit of such measures are themselves cost-effective.

### Austerity and community vulnerability to GI infections

Our findings are particularly pertinent to questions of community vulnerability to GI infections [34, 45]. As austerity compromises the work of EHOs, particularly in Stoneville, via fewer ‘boots on the ground’, many food businesses are operating unchecked or without timely advice, making them less well-equipped to serve their customers safe food. Limiting ERS preventative functions erodes the ability of public protection teams to protect their local communities from GI infections. This protection is further challenged as austerity shapes the prioritisation of GI infection work, and intervening in high-risk infections must be prioritised over lower-risk infections in the areas most affected by budget cuts. We argue that the decreasing capacity of EHOs as boots on the ground, reductions in preventative functions and changing prioritisation of work have increased community vulnerability to GI infections in Stoneville more than in Meadowshire.

Our findings suggest that political rationing of resources due to austerity measures may be leading to socio-spatial inequalities in ERS provision, subsequent GI infection prevention and control and unfair and unjust inequalities in community vulnerability, shaped by unequal cuts and losses of staff. These inequalities in community vulnerability can be directly attributed to policy decisions to cut local authority budgets in pursuit of austerity measures. In connecting government policy to increasing community vulnerability to GI infections in this way, our understanding sits in stark contrast to much GI infection literature which often sees vulnerability as linked to individual biology or behaviours [32, 38]. Illuminating these structural factors is vital because it removes the inclination to blame individual ‘disadvantaged’ communities for illness and renders community vulnerability to GI infections amenable to policy change.

### Strengths and limitations

Being a qualitative study limits generalisability beyond our study areas but its findings may apply to local authorities with similar contexts.

Collecting data during the COVID-19 pandemic limited our time conducting fieldwork observations. To minimise the impact of the study on participants’ pandemic work, we did not recruit during peak COVID waves and, once restrictions on in-person data collection was lifted, limited our observations with EHOs. The pandemic also prevented us from recruiting local authorities at the extreme ends of ERS budget cuts as, understandably, not all local authorities we approached were amenable to taking part in research at this time. Notwithstanding, our research shows how extreme differences in budget cuts are not required to see a significant difference to the lived experiences of providing GI infection work. Additionally, collecting data during the pandemic when GI infections were at a historical low [37], reinforces evidence for the continued impact of austerity on GI infection work across our study areas.

Where possible, participants were asked to give examples of GI work in Stoneville and Meadowshire. When it was clear that participants working across large regions (e.g. UKHSA staff) were referencing general experiences, these quotes were used to draw out general themes in terms of crucial aspects of local health protection work and the impacts of austerity, rather than the specific context of Stoneville or Meadowshire. We have since presented our work to other local health protection teams and the UKHSA and found that our findings resonate with experiences beyond Stoneville and Meadowshire.

## Conclusion and policy implications

Recent years have seen increasing threats from infectious disease outbreaks such as COVID-19, Measles and Pertussis in the UK [26, 66]. EHOs can play an important role in managing these outbreaks. This was demonstrated during the COVID-19 pandemic when eight out of ten EHOs working for local authorities were redeployed [15]. Although our findings are specific to GI infections, they may, therefore, also be relevant to wider infectious disease control.

Recruiting more EHOs in areas which have experienced the greatest cuts could be one way to tackle infectious disease outbreaks and mitigate against inequalities. The impact of austerity in our study was not restricted to public protection teams but was also felt across other parts of the local health protection system that relied on EHO work. This suggests that ring-fencing budgets to only certain parts of local health protection systems to protect services, as has been done for local authority public health teams [25] might be ineffective unless the *whole* local health protection system is being shielded from cuts. If other parts of local health protection systems such as Directors of Public Health and the UKHSA advocated for the protection of public protection teams, this could help manage the workloads of their own staff, as well as support EHO colleagues.

Going forward, we need to understand the impacts that lost services are having on GI infections so evidence-based approaches can be (re)introduced. Meanwhile, staff who are increasingly forced to ration and prioritise their work according to risk should be supported and trained in risk assessment tools and methods. Indeed, the Food Standards authority, in response to pressures within public protection teams, have, since this study was conducted, published updated Food Law Codes of Practice which prioritises controls at ‘high risk’ food establishment [20, 21]. While this seems to have been broadly welcomed by professionals working in public protection, it is important that EHOs are supported in applying these new principles alongside other risk assessments required in other aspect of their work. If risk assessment is poorly applied (due to under-resourcing) it may exacerbate GI infection work in an already strained system.

Prevention is an upstream public health intervention, a core part of health protection, and essential for reducing health inequalities [67]. However, the prioritisation of reactive work over preventative functions in this study is echoed across other aspects of local authority services affected by cuts, showing that preventative work can be one of the first services to be lost when resources are constrained [56, 63]. Action is needed to ensure that austerity measures do not force health protection to become a reactive service, more in line with clinical medicine, than staying true to its public health, preventative roots.

## Supplementary Information

Supplementary Material 1

Supplementary Material 2

Supplementary Material 3

## Data Availability

Data not available. Due to the potentially politically sensitive nature of the research, the participants of this study did not give consent for their data to be shared. This is to protect the privacy of individuals, and the identity of the local authorities included in the study. Supporting data is not therefore available.

## Abbreviations

COVID-19: Coronavirus disease
EHO: Environmental Health Officer
ERS: Environmental and Regulatory Services
GI infection: Gastrointestinal infection
IPCT: Infection Prevention and Control Team
LAPHT: Local Authority Public Health Team
PPT: Public Protection Team
STEC: Shiga toxin-producing *E*. *coli*
UK: United Kingdom
UKHSA: UK Health Security Agency

## Footnotes

1 We place the terms ‘advantaged’ and ‘disadvantaged’ in inverted commas throughout this paper to reflect the understanding that, while these categorisations are terms commonly used within the literature they are socially constructed and may not be recognised by the people living in those places [12, 48].

2 The authors of this paper understand ‘risk’ as a contested term and one which is socially constructed [19, 24]. However, when we use the term ‘risk’ in these results, we use it in the way our participants understood it i.e. as the potential for harm to the population.

3 See Williams and Fullagar [67] for an explanation of this concept [67].
