# Supplementary material for: Increasing community vulnerability to gastrointestinal infections in austerity’s shadow: a comparative study of two English local authorities
Source: BMC Public Health. 2026 Mar 5;26:1198. doi: 10.1186/s12889-026-26657-1 (PMC13072539; doi:10.1186/s12889-026-26657-1)
Supplement: Supplementary file 1 — Supplementary Material 1 [file 12889_2026_26657_MOESM1_ESM.docx]

**Appendix A: GRIPP2-Short Form**

| Section and Topic | Item | Reported on page no. |
| --- | --- | --- |
| 1. Aim | Patient and Public Involvement aimed to involve the public in all stages of the research project. | 8 |
| 1. Methods | Ten members of the public were consulted across all stages of the project. These members of the public were recruited from the NIHR HPRU in GI infections PPI group <http://hprugi.nihr.ac.uk/> and the ARC NW Coast PPI group <https://arc-nwc.nihr.ac.uk/>. At the methods stage, PPI members were involved in:   - Refining the focus of the project - Advising on recruitment strategies. - Highlighting the importance of providing clear definitions around austerity. - Suggesting which measures of government spending cuts could be used to choose areas. | 7-8  9  5  7 |
| 1. Study results | PPI contributed to the study in several ways, including:   - Refining data analysis - Highlighting some of the limitations of the study. | 12-25  27-28 |
| 1. Discussion and Conclusions | Patient and Public Involvement in this study was effective and influenced important aspects of the study (see sections 2 & 3). |  |
| 1. Reflections/Critical perspectives | It was beneficial that public partners were already involved in several other studies so had experience in this type of work. As SR has involved a number of these partners in other projects, this has helped the relationships between public partners and the research team develop and has increased trust in the collaboration process.  Involving the same partners from the beginning to the end of the study to the end allowed the public partners to contribute in several ways. This was facilitated by funding for partners' time through the NIHR HPRU-GI.  There were some limitations. Partners were not actively involved in recruiting participants or coding the qualitative data. PPI was embedded into many aspects of this study but with further thought and training could have been embedded further e.g. with PPI training in thematic coding.  Partners appreciated good communication from the research team and stressed the importance of being informed about where and how their contributions had made a difference to the study. |  |
