# Supplementary material for: Increasing community vulnerability to gastrointestinal infections in austerity’s shadow: a comparative study of two English local authorities
Source: BMC Public Health. 2026 Mar 5;26:1198. doi: 10.1186/s12889-026-26657-1 (PMC13072539; doi:10.1186/s12889-026-26657-1)
Supplement: Supplementary file 2 — Supplementary Material 2 [file 12889_2026_26657_MOESM2_ESM.docx]

| **Main Theme** | **Subthemes** | **No. of participants coded to this theme/subtheme** |
| --- | --- | --- |
| Boots on the ground | Onsite expertise:  physical inspection of food premises | 15 |
|  | Accessibility to the public:  responding to public concerns | 8 |
| Local Health Protection System Integration |  | 17 |
| Managing ‘risk’ | Using assessments of ‘risk’ to prioritise work | 15 |
|  | Preventative work | 16 |

**Appendix B: Table of Themes**
